# Supplementary material for: Surface-based tracking for short association fibre tractography
Source: Neuroimage. 2022 Oct 15;260:119423. doi: 10.1016/j.neuroimage.2022.119423 (PMC10009610; doi:10.1016/j.neuroimage.2022.119423)
Supplement: Supplementary file 1 [file mmc1.docx]

# Appendix A. Acceptance/rejection of streamlines by GG filter


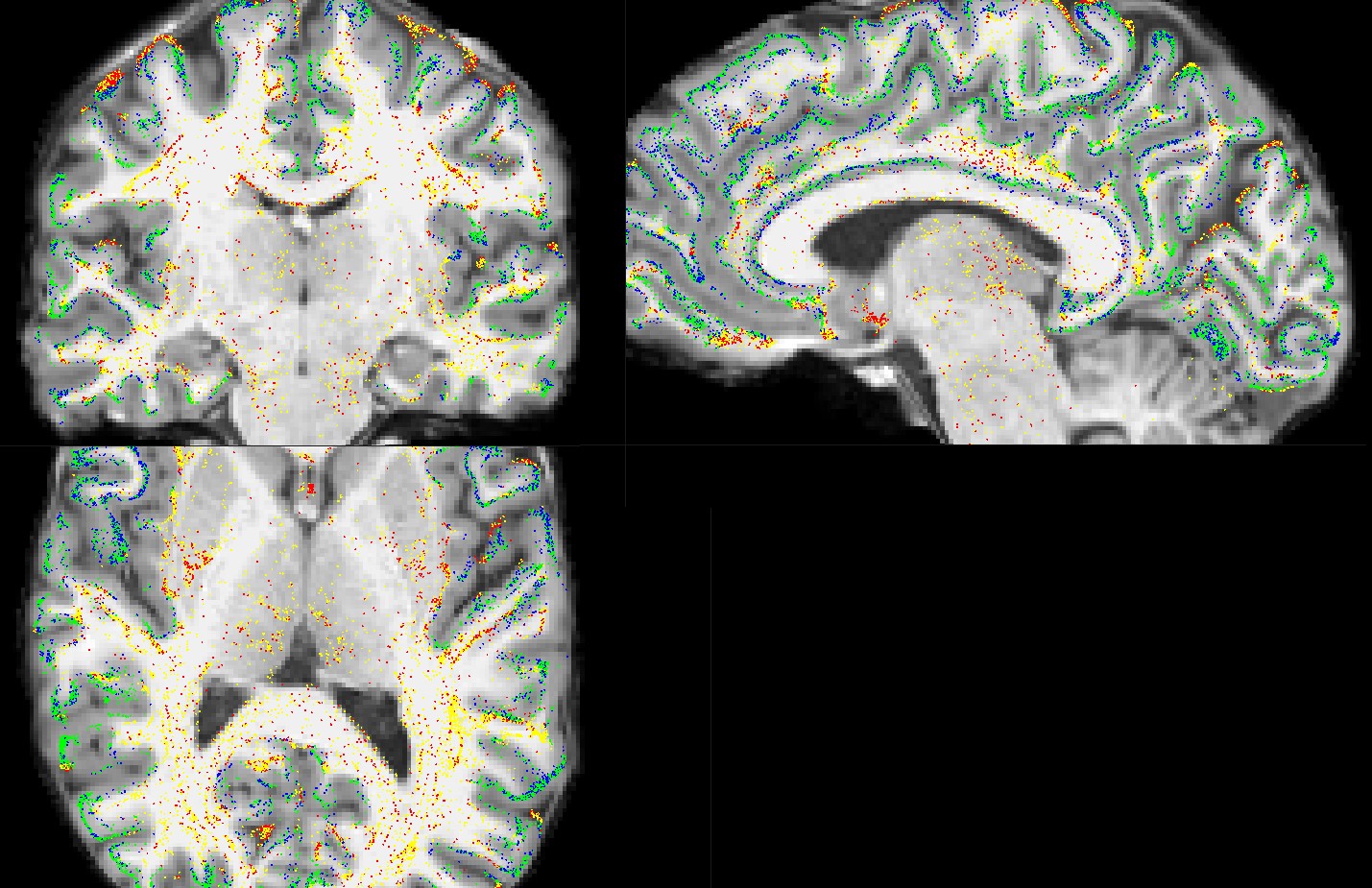


Figure A.1: Both ends of each streamline are demonstrated as points overlaid on T1 image (in dMRI space) in three orthogonal planes. Ends of accepted streamlines are shown in green; all other colours represent rejected streamlines: those with both ends rejected (red) and those with one end rejected (yellow - rejected end; blue - other end). All ends classed by the filter as residing within the cortex (green and blue points) are seen in expected areas; the vast majority of ends rejected by the filter (red and yellow points) are in the white matter, subcortical grey matter, cerebellum or CSF spaces. It is possible that a small proportion of rejections was due to the imperfections of T1-DWI registration. In this early demonstration, 1M seeds were used with no upper limit to streamline length.
