# Supplementary material for: Surface-based tracking for short association fibre tractography
Source: Neuroimage. 2022 Oct 15;260:119423. doi: 10.1016/j.neuroimage.2022.119423 (PMC10009610; doi:10.1016/j.neuroimage.2022.119423)
Supplement: Supplementary file 4 [file mmc4.docx]

**Appendix D. Example of streamline data representation of the surface in a single subject**


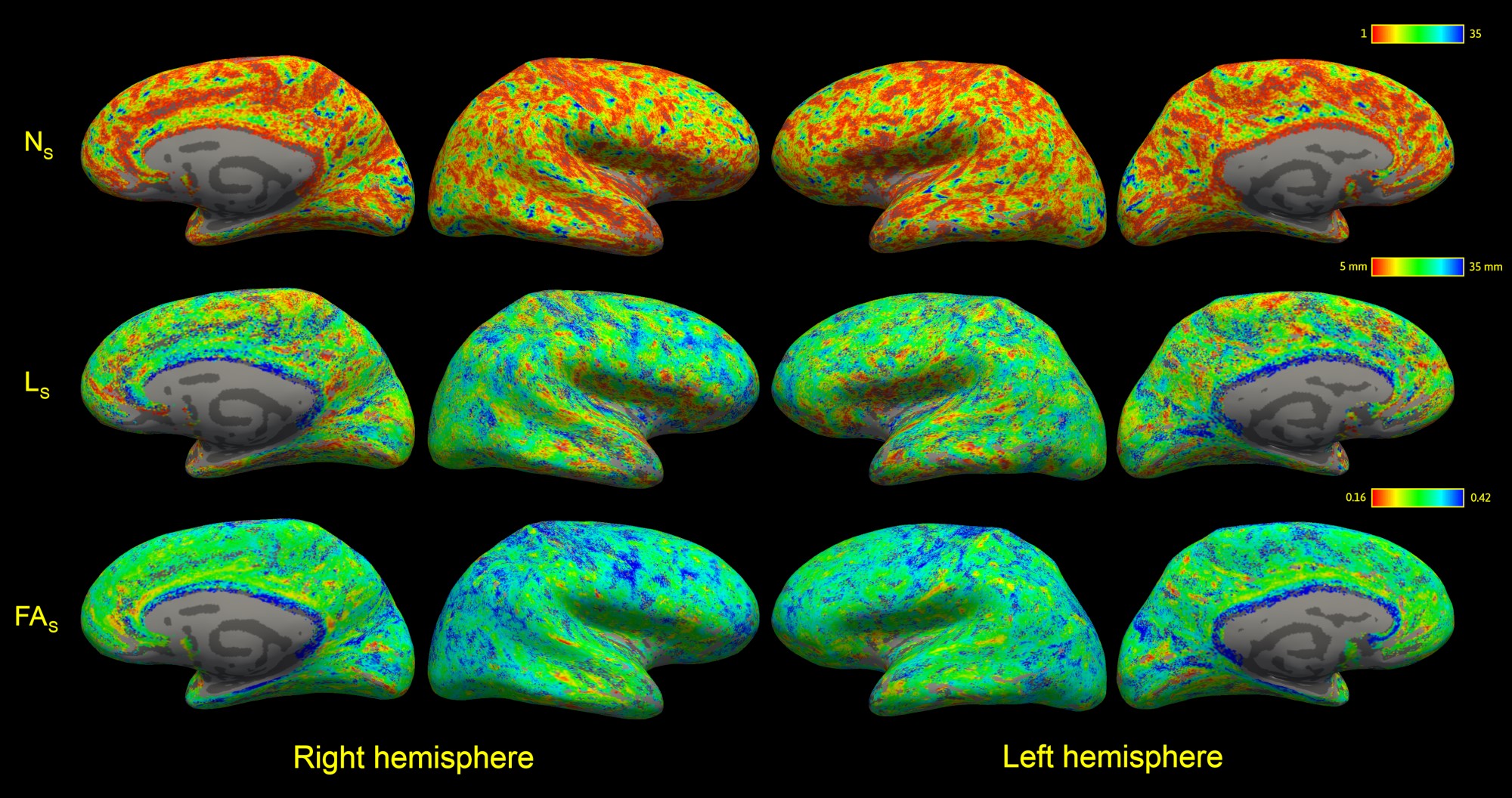


Figure D.1: Data shown are projected on the subject surface, with no smoothing applied. N_S_, termination density (number of streamlines/vertex). L_S_, mean streamline length/vertex. FA_S_, mean streamline fractional anisotropy/vertex.
