# Supplementary material for: Surface-based tracking for short association fibre tractography
Source: Neuroimage. 2022 Oct 15;260:119423. doi: 10.1016/j.neuroimage.2022.119423 (PMC10009610; doi:10.1016/j.neuroimage.2022.119423)
Supplement: Supplementary file 5 [file mmc5.docx]

# Appendix E. Misalignment between differently resampled data


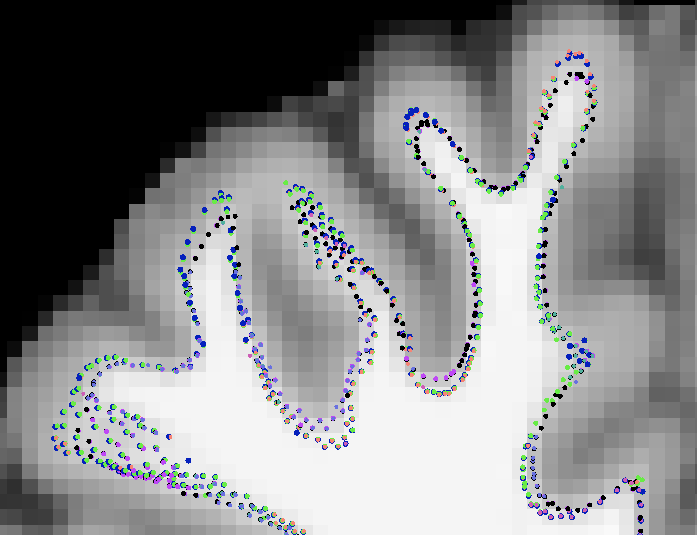
Figure E.1: Plot of white surface mesh vertices from all data sets together in the same DWI space after registration, overlaid on T1-weighted image. The same mesh coordinates were brought into the corresponding diffusion spaces (for surface-based tractography) which were then brought into the space of Prisma/SA/2mm isotropic data set (shown here) using FA volume co-registration. Most misalignment occurs in sulcal fundi and gyral crowns. These imperfections are exclusively between data sets with different voxel sizes: black, purple, lilac, cyan points show seeds from the 1×1×1 mm^3^ data while navy blue, light green, rose, pink (barely seen) points show seeds from the 2×2×2 mm^3^ data. The sulcal wall demonstrating the most difference in Figure 4 (yellow arrow) appears reasonably aligned.
