# Supplementary material for: Surface-based tracking for short association fibre tractography
Source: Neuroimage. 2022 Oct 15;260:119423. doi: 10.1016/j.neuroimage.2022.119423 (PMC10009610; doi:10.1016/j.neuroimage.2022.119423)
Supplement: Supplementary file 6 [file mmc6.docx]

**Appendix F. Consistency of SAF demonstrated using surface-based analysis**


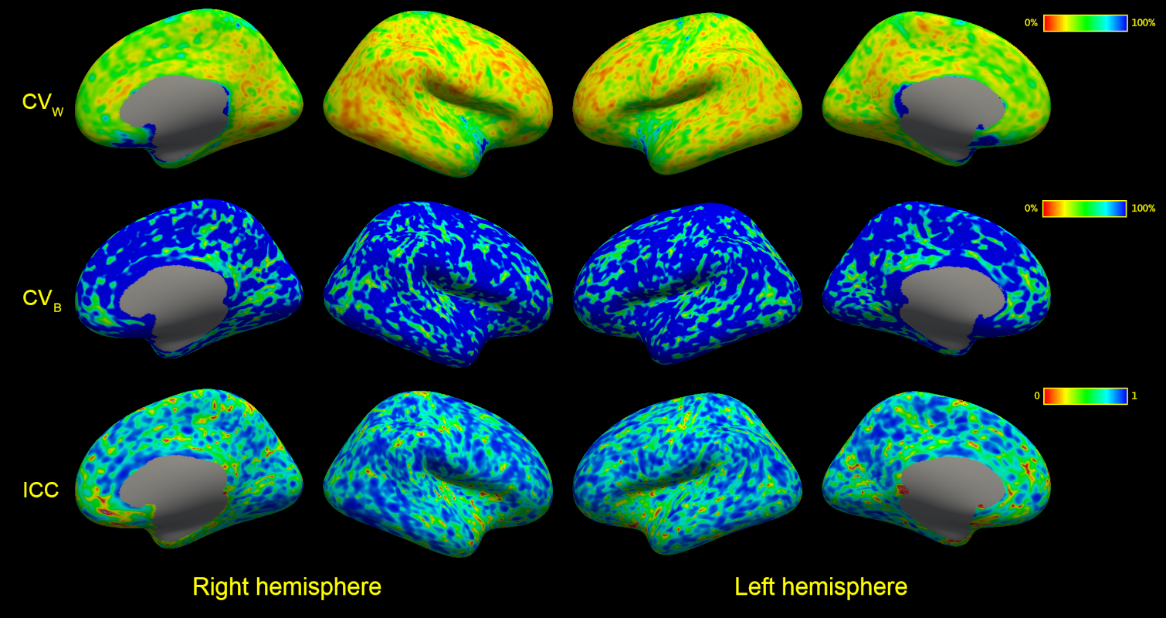


Figure F.1: Number of streamlines per vertex. CV_W_, coefficient of variation within subjects. CV_B_, coefficient of variation between subjects. ICC, intraclass correlation coefficient. Values at each vertex were recorded in subject space, then transformed into average subject space before running analyses. CV_W_ and CV_B_ were thresholded at 100%.


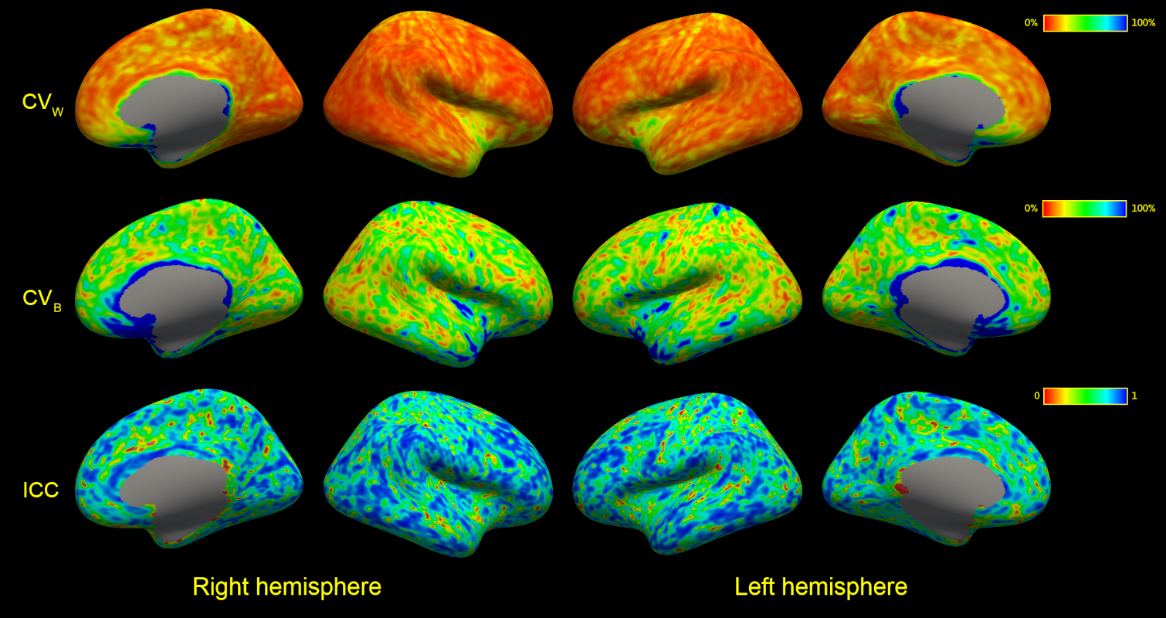


Figure F.2: Mean length of streamlines per vertex. CV_W_, coefficient of variation within subjects. CV_B_, coefficient of variation between subjects. ICC, intraclass correlation coefficient. Values at each vertex were recorded in subject space, then transformed into average subject space before running analyses. CV_W_ and CV_B_ were thresholded at 100%.


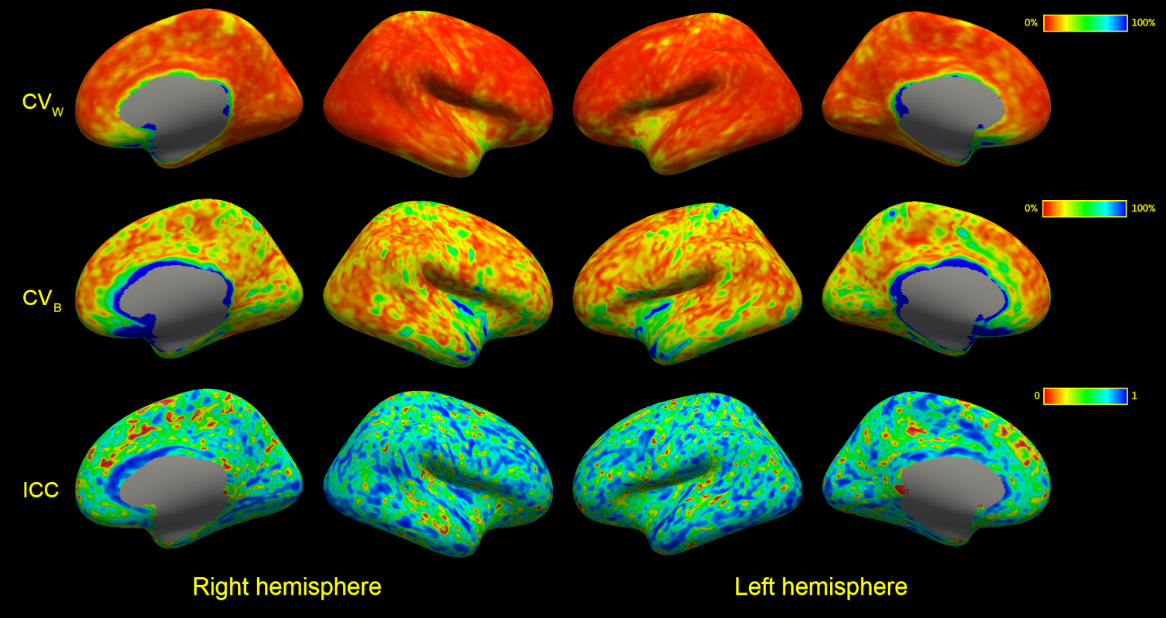


Figure F.3: Mean fractional anisotropy along streamlines per vertex. CV_W_, coefficient of variation within subjects. CV_B_, coefficient of variation between subjects. ICC, intraclass correlation coefficient. Values at each vertex were recorded in subject space, then transformed into average subject space before running analyses. CV_W_ and CV_B_ were thresholded at 100%.
